# In Situ Imaging Reveals Efficient Charge Separation in Monolayer MoS_2_–WS_2_ Type-II Heterojunctions

**DOI:** 10.1021/jacs.5c19244

**Published:** 2026-02-20

**Authors:** Qing Huang, Ziyuan Wang, Rujia Liu, Hanyu Yao, Chenwei Ni, Tianyu Bo, Shu Wu, Fusai Sun, Fengtao Fan, Michael V. Mirkin

**Affiliations:** † State Key Laboratory of Catalysis, Dalian National Laboratory for Clean Energy, iChEM, 58279 Dalian Institute of Chemical Physics, Chinese Academy of Sciences, Zhongshan Road 457, Dalian 116023, China; ‡ 74519 University of Chinese Academy of Sciences, Beijing 100049, China; § Department of Chemistry and Biochemistry, 14781 Queens College-CUNY, Flushing, New York 11367, United States; ∥ College of Chemistry, Beijing Normal University, Beijing 100875, China; ⊥ The Graduate Center of CUNY, New York, New York 10016, United States

## Abstract

Covalently bonded in-plane two-dimensional (2D) transition metal dichalcogenide (TMD) heterojunctions with atomically sharp interfaces hold great promise for photocatalytic applications in solar energy conversion and environmental remediation; however, their spatially resolved charge distribution and transport, particularly under operando conditions, remain poorly understood. Here, we employ photoscanning electrochemical microscopy (photo-SECM) to directly visualize photoinduced charge separation in monolayer MoS_2_–WS_2_ in-plane heterojunctions. Spatial separation of photogenerated carriers is observed, with electrons accumulating in MoS_2_ and holes in WS_2_, leading to strongly asymmetric interfacial kinetics: Fc^+^ reduction proceeds rapidly on MoS_2_ (0.6 cm s^–1^), whereas Fc oxidation on WS_2_ is significantly slower (0.008 cm s^–1^). High-resolution surface photovoltage microscopy (SPVM) enables a quantitative comparison of charge-separation capacity across architectures. The in-plane MoS_2_–WS_2_ heterojunction shows the largest photovoltage contrast (−35 mV in MoS_2_, 20 mV in WS_2_), exceeding the vertical heterojunction (−18 mV in MoS_2_, 11 mV in WS_2_) and the individual monolayers (−12 mV for MoS_2_, – 1 mV for WS_2_), establishing the following trend: in-plane > vertical > monolayers. Ultraviolet photoelectron spectroscopy (UPS) indicates that this directional charge separation is driven by intrinsic type-II band alignment, while photoluminescence (PL) imaging shows that the interface acts as a recombination center that limits efficient carrier extraction. These results provide direct experimental evidence of type-II-driven charge separation in in-plane heterojunctions and offer critical insights for interface design in high-efficiency photocatalytic and optoelectronic systems.

## Graphical abstract

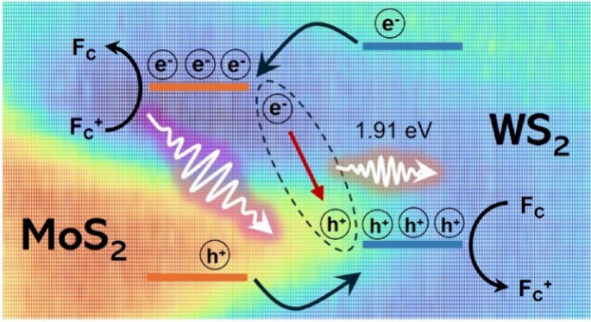

## Introduction

Photocatalysis is a major technological pathway for harnessing solar energy and sustainable production of clean chemical fuels.^1^ In practical photocatalytic processes the charge separation is often a critical factor determining the overall performance.^2^ Heterojunction structures can facilitate effective charge transfer between different semiconductor components, thus prolonging the lifetime of photogenerated carriers and improving their spatial separation.^3^ In particular, TMD heterojunctions showed superior light absorption, enhanced carrier mobility, and favorable band alignments arising from synergistic interactions between different materials.^4,5^ In vertically stacked MoS_2_–WS_2_ heterojunctions, charge transfer occurs primarily through interlayer tunneling between monolayers. Wang’s group, using femtosecond pump–probe spectroscopy, observed that holes can transfer from MoS_2_ to WS_2_ within 50 fs.^6^ Sambur and co-workers used photocurrent microscopy, to find that the charge transport strongly depends on the stacking order of MoS_2_ and WS_2_.^7^ Despite rapid interlayer transfer, the tunneling barrier in vertical heterojunctions inherently limits the overall mobility and spatial freedom of charge carriers.

Unlike vertical heterojunctions, in-plane TMD heterojunctions form atomically sharp, seamless interfaces that eliminate interlayer tunneling barriers and allow electrons and holes to move laterally with faster charge-transfer rates.^8,9^ Theoretical studies, including time-dependent density functional theory and nonadiabatic molecular dynamics, revealed complex interactions between charge-transfer excitons and interfacial recombination.^10–12^ Visualizing carrier distribution and participation in chemical reactions is therefore essential for understanding charge transport and designing efficient photocatalysts.

Among a few experimental tools currently available for studying in-plane heterojunctions,^13^ photo-SECM enables high-resolution in-operando imaging of photocatalytic activity.^14–20^ We have recently employed the feedback mode of photo-SECM to visualize photogenerated electrons and holes on the nanoscale and directly correlate measured photocurrents with the spatial separation of charge carriers.^20^ In this work, we investigate the carrier dynamics in epitaxially grown MoS_2_–WS_2_ lateral heterojunctions to elucidate how interfacial properties govern charge separation in in-plane 2D heterojunctions. By combining photo-SECM, surface photovoltage microscopy (SPVM), and photoluminescence (PL) spectroscopy, we directly visualize and quantify both charge separation and recombination processes. Our results reveal efficient directional carrier separation, with electrons predominantly accumulating in the MoS_2_ domain and holes in the WS_2_ domain, driven by the intrinsic type-II band alignment. During charge transfer, a fraction of electrons and holes undergo interfacial recombination, which partially limits the further enhancement of charge separation efficiency. Furthermore, a comparative study between vertical and lateral MoS_2_–WS_2_ heterojunctions shows that lateral heterojunctions exhibit markedly superior carrier separation efficiency.

## Results and Discussion

MoS_2_–WS_2_ lateral heterojunctions were synthesized via chemical vapor deposition (CVD; see Supporting Information Methods and Figure S1). Optical microscopy clearly distinguishes the inner MoS_2_ and outer WS_2_ domains, as well as the lateral interface (Figure S2). atomic force microscopy (AFM) measurements confirm that both domains are monolayers (Figure S3). Raman spectroscopy further verifies the material composition, showing characteristic E′ and A_1_′ modes of MoS_2_ in the inner region and A_1_′, 2LA­(M), and E′ modes of WS_2_ in the outer region (Figure S4). Raman intensity mapping based on the MoS_2_ E′ mode at 381 cm^–1^ and the WS_2_ E′ mode at 351 cm^–1^ clearly demonstrates the formation of an in-plane MoS_2_–WS_2_ heterojunction, with a triangular monolayer MoS_2_ domain as the core and WS_2_ as the shell layer (Figure S5). The heterojunction interface was examined by high-resolution scanning transmission electron microscopy (STEM) Z-contrast imaging (Figure S6). Due to significantly higher image intensity of W atoms compared to that of Mo atoms, sharp zigzag step interfaces can be clearly resolved.^21,22^ The WS_2_ and MoS_2_ domains are observed to connect seamlessly at the interface, forming a continuous single hexagonal monolayer lattice with a shared crystallographic orientation.

Photo-SECM was used to probe the photoinduced charge distribution (Figure 1A). In a feedback mode experiment, ferrocenemethanol (Fc) served as the redox mediator, and the SECM tip potential (*E*_T_) was held at 0.4 V, i.e., sufficiently positive to oxidize Fc. When the tip (the tip radius, *a* = 260 nm) was scanned laterally over the heterostructure (the white arrow in the inset of Figure 1B) the tip current variation in the dark between MoS_2_ and WS_2_ was <2 pA (black curve in Figure 1B), and no measurable reduction of Fc^+^ occurred at either surface (“negative feedback”).

**Figure 1:**
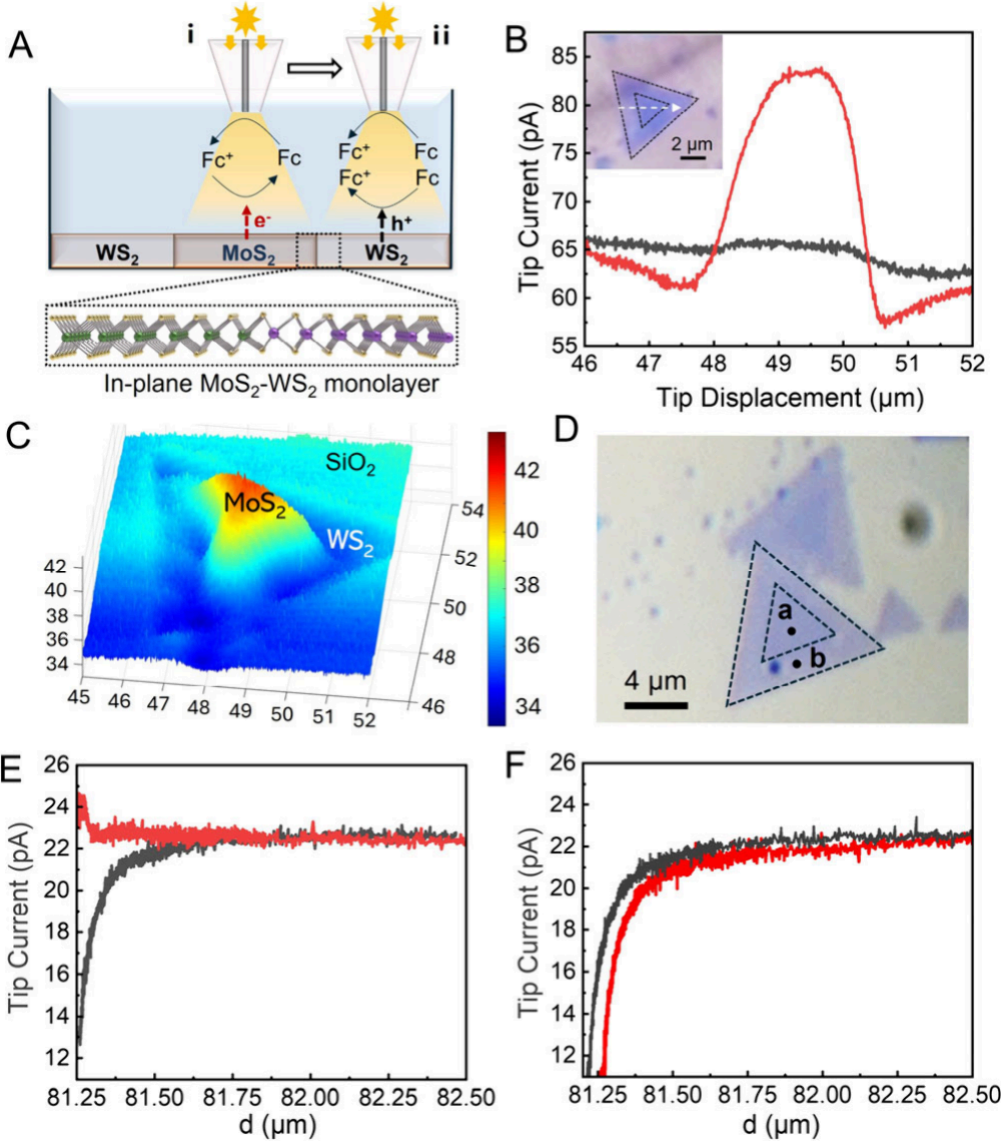
Photo-SECM of in-plane MoS_2_–WS_2_ heterojunction. (A) Schematic of the photo-SECM experiments in feedback (i) and redox competition (ii) modes with through-tip illumination of the sample. (B) Lateral scans along the specified trajectory (white line in the inset) under illumination (red curve) and in the dark (black). (C) Constant-height photo-SECM image of the same heterojunction. (D) Optical micrograph of the in-plane MoS_**2**_–WS_**2**_ heterojunction. (E and F) Approach curves obtained over locations **a** and **b** shown in panel D, respectively, in the dark (black curves) and under illumination (red curves). Solution contained 1 mM Fc in 0.1 M phosphate buffer (pH 7). *E*_T_ = 0.4 V vs Ag/AgCl. *a*, nm = 260 (B), 150 (C), and 70 (E, F).

Under illumination (red curve in Figure 1B; the tip/substrate distance, *d* ≈ 500 nm, was the same as in the dark scan), the tip current (*i*_T_) significantly increased over the MoS_2_ surface due to the regeneration of Fc (positive feedback; Figure 1Ai). When the tip was scanned over illuminated WS_2_ surface, the *i*_T_ values were lower than those measured over an insulating surface (cf. red and black curves in Figure 1B) due to redox competition,^23^ i.e., the same Fc oxidation reaction occurring at the tip and WS_2_ surfaces (Figure 1Aii). This behavior is most pronounced at the portions of WS_2_ surface adjacent to the heterojunction. These observations suggest that, under illumination, photogenerated electrons migrate to the MoS_2_ surface where they reduce Fc^+^ to Fc, whereas photogenerated holes accumulate on the WS_2_ surface and oxidize Fc to Fc^+^, thus competing with the tip’s process and causing the *i*_T_ decrease below the negative feedback level (Figure 1Aii). In the high-resolution photo-SECM image (Figure 1C; *a* = 150 nm), the inner region (MoS_2_) exhibits a higher photocurrent due to Fc regeneration, whereas a lower current (negative feedback) is observed over the insulating SiO_2_ support, and the lowest *i*_T_ (redox competition) corresponds to WS_2_ surface.

The approach curves (Figure 1E,F; *a* = 70 nm) were recorded, respectively, over the MoS_2_ and WS_2_ regions (locations **a** and **b** in Figure 1D) either in the dark (black curves) or under illumination (red curves). Negative feedback at point **a** in the dark suggests that either the conductivity of MoS_2_ or its ability to regenerate Fc is low, and positive feedback under illumination points to the reduction of Fc^+^ by photogenerated electrons on the MoS_2_ surface. At point **b**, the transition from negative feedback in the dark (black curve in Figure 1F) to redox competition response under illumination (red curve) suggests that photogenerated holes accumulate on the WS_2_ surface and oxidize Fc.

Finite element simulations using a two-region model corresponding to the heterojunction geometry were performed to evaluate kinetic parameters for electron transfer at MoS_2_ and WS_2_ surfaces. In these simulations, the oxidation of Fc at the tip is diffusion controlled, the Fc^+^ reduction occurs under illumination on the inner triangular region, and Fc oxidation—on the periphery representing WS_2_, whereas in the dark the entire substrate is inert. The tip radius (*a* = 70 nm) and the ratio of glass radius to that of the conductive disk of the tip (RG = 2.5) were determined by fitting the experimental approach curve recorded over MoS_2_ in the dark (Figure 2A, black dots) to the simulated curve (red). Using these parameters, the approach curves obtained under illumination over MoS_2_ (Figure 2B, black) and WS_2_ (Figure 2C, black) were fitted by to the theory (red), yielding the rate constants of 0.6 cm/s for Fc^+^ reduction on MoS_2_ and 0.008 cm/s for Fc oxidation on WS_2_. While these values for two half-reactions are not directly commensurate, the strong asymmetry demonstrates photocarrier-selective interfacial charge transfer across the MoS_2_–WS_2_ junction, where photogenerated electrons and holes preferentially populate MoS_2_ and WS_2_, respectively. The corresponding Fc concentration distribution at the substrate surface is shown in Figure 2D, and the simulated lateral tip scan along the dashed line (Figure 2E) reproduces the key features of the experimental line scan in Figure 1B.

**Figure 2:**
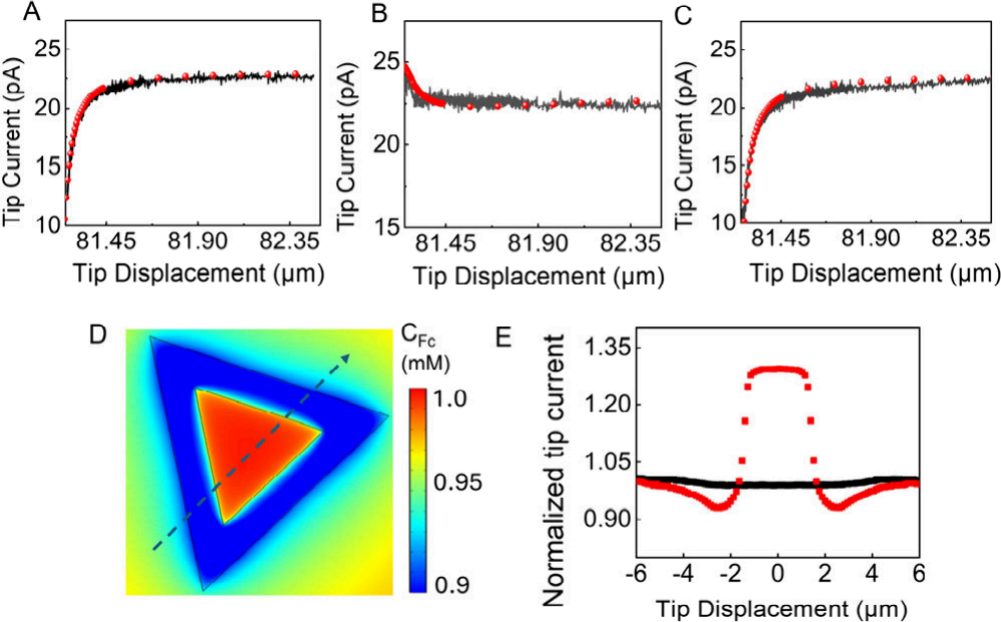
COMSOL simulations and photo-SECM experiments at an in-plane MoS_2_–WS_2_ heterojunction. (A–C) Experimental (black dots) and simulated (red circles) approach curves at MoS_**2**_ surface in the dark (A) and MoS_**2**_ (B) and WS_**2**_ (C) surfaces under illumination. (D) Simulated Fc concentration distribution. (E) Simulated lateral tip scans across the heterojunction with (red) and without (black) illumination. *a* = 70 nm, RG = 2.5, D_Fc_ = 7.9 × 10^–6^ cm^**2**^/s, *D*_Fc+_ = 4.5 × 10^–6^ cm^2^/s.

Additional evidence of charge separation occurring at the WS_2_–MoS_2_ interface comes from photo-SECM mapping of partially broken heterojunctions (Figure 3). While normally WS_2_ and MoS_2_ layers are seamlessly connected along the triangle edges (see heterojunctions inside blue squares in Figure 3A and corresponding schematic in Figure 3Bi), some heterojunctions are partially broken due to mechanical stress (labeled by orange squares in Figure 3A). In such fractured heterojunctions, only the three vertices of a MoS_2_ triangle remain in contact with WS_2_ (blue circles in Figure 3Bii), forming discrete junctions. A photo-SECM image of a partially broken heterojunction (Figure 3C) reveals that only small portions of the WS_2_ surface near these vertices exhibit significant Fc photooxidation, whereas the portions of WS_2_ separated from MoS_2_ show negligible activity. A lateral scan along the dashed line in the inset of Figure 3C shows modest positive feedback over MoS_2_ and redox competition signal over WS_2_ under illumination (Figure 3D). These findings underscore the critical importance of a continuous and well-formed interface for effective charge separation in in-plane heterojunctions.

**Figure 3:**
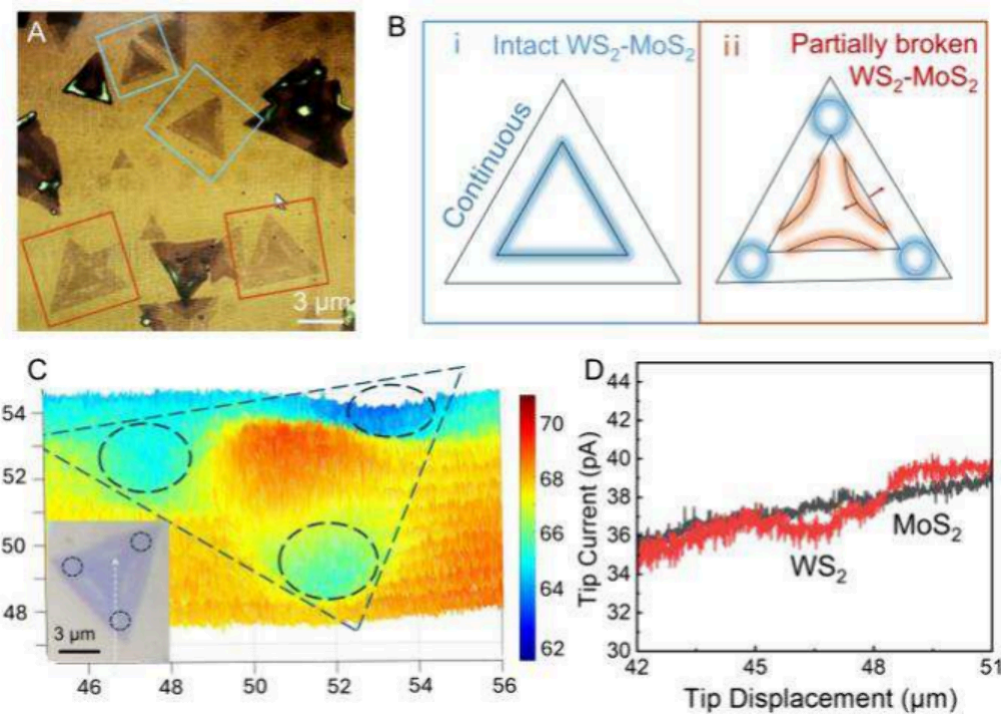
Photo-SECM of partially broken heterojunctions. (A) Optical micrograph and (B) schematic diagrams of intact (blue squares) and partially broken (orange squares) Mo_2_–WS_2_ heterojunctions. (C) Constant-height photo-SECM image and optical micrograph (inset) of the same fractured heterojunction. (D) Lateral scans along the same trajectory indicated by the dashed line in the inset of panel C, recorded under illumination (red) and in the dark (black). Dark triangles in panel A are MoS_2_ multilayers. Solution contained 1 mM Fc in 0.1 M phosphate buffer (pH 7). *E*_T_ = 0.4 V vs Ag/AgCl. *a*, nm = 280 (C) and 148 (D).

The intrinsic charge separation behavior of the MoS_2_–WS_2_ heterojunction was investigated using SPVM, where the SPV magnitude directly reflects the distribution of photogenerated charge concentration.^24^ The SPVM image (Figure 4B) was obtained by subtracting the surface potential in the dark (Figure S9A) from that under illumination (Figure S9B). Figure S10 presents the specific SPV values measured along the white dashed line indicated in Figure 4B. It can be seen that in air, photogenerated electrons accumulate in MoS_2_, while photogenerated holes accumulate in WS_2_. This is consistent with the charge separation phenomenon observed in the liquid phase. We further performed statistical analysis on other heterojunctions (Figure S11), which showed a highly reproducible charge separation behavior. The mean SPV values of MoS_2_ and WS_2_ in the in-plane heterojunctions were determined to be −35 mV and 20 mV, respectively. Much smaller SPV values were obtained from SPVM images of individual MoS_2_ (Figure S12) and WS_2_ (Figure S13) triangles, i.e., −12 mV and −1 mV, respectively, and confirmed by chopped-light measurements (Figure S14). These results confirm that the formation of the heterostructure significantly enhances charge separation.

**Figure 4:**
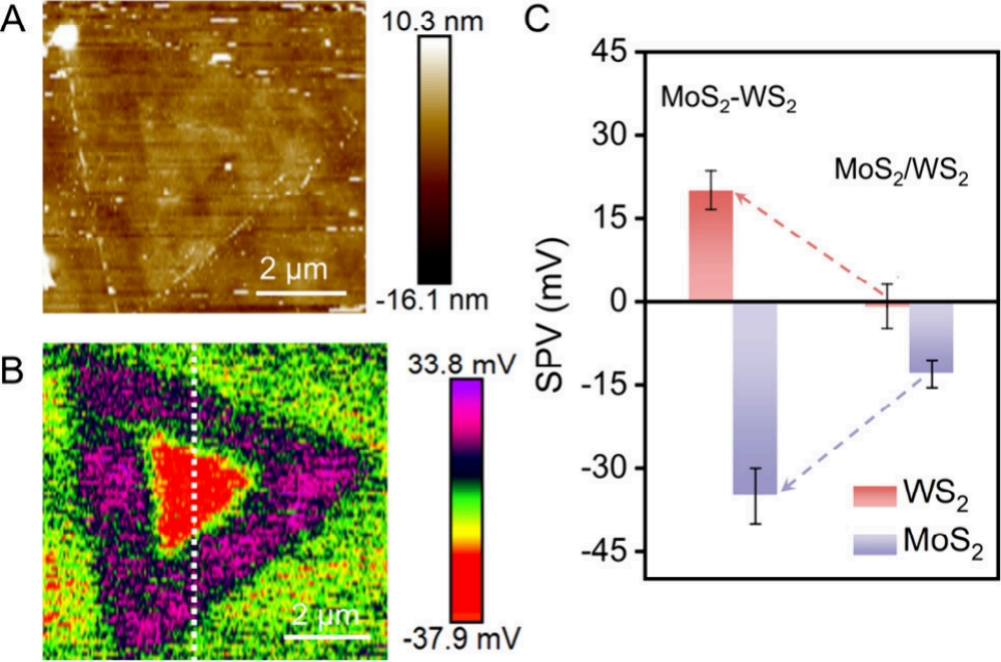
SPVM of in-plane MoS_2_–WS_2_ heterojunction. (A) AFM topography image of in-plane MoS_2_–WS_2_ heterojunction. (B) SPVM image of in-plane MoS_2_–WS_2_ heterojunction. (C) Comparison of the mean SPV values of in-plane MoS_2_–WS_2_ heterojunctions (Figures 4B and S11) to those measured in separate MoS_2_ and WS_2_ (Figures S12–S14). The error bars are the standard deviations of SPV values.

The charge recombination behavior in the in-plane MoS_2_–WS_2_ heterojunction was investigated using PL spectroscopy. Figure 5A shows the optical microscopy image of the heterojunction used for PL measurements. With a laser spot size of approximately 1 μm^2^, PL spectra collected from the outer region (point 1 in Figure 5A) and the inner region (point 3 in Figure 5A) exhibit characteristic emission peaks of WS_2_ at 630 nm and MoS_2_ at 685 nm, corresponding to A-exciton resonances at 1.97 and 1.81 eV, respectively.^8,25^ The corresponding PL intensity maps (Figure S15) further demonstrate that the emissions at 1.97 and 1.81 eV are spatially localized in the WS_2_ and MoS_2_ regions, respectively, in excellent agreement with the spatial distribution of MoS_2_/WS_2_ domains in the heterojunction.

**Figure 5:**
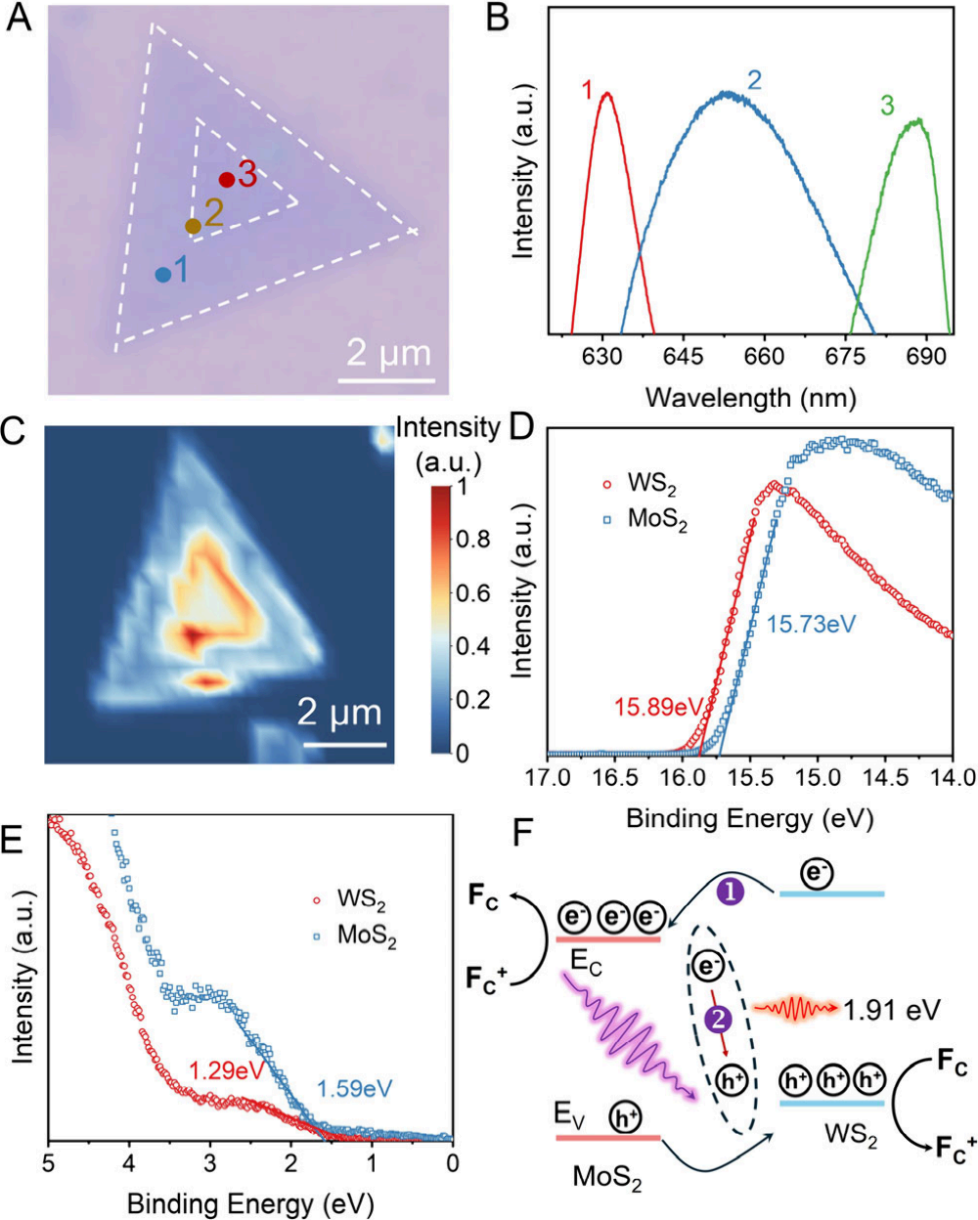
UPS and PL characterizations of in-plane Mo_2_/WS_2_ heterojunction. (A) Optical micrograph of a triangular in-plane MoS_2_–WS_2_ heterojunction. (B) PL spectra of the points marked by 1–3 in panel A. The peak positions for spectra 1 and 3 are 630 and 685 nm, respectively, indicating WS_2_ and MoS_2_ materials; a peak at the interface (region 2) is at 650 nm. (C) PL intensity mapping at 650 nm. (D, E) Zoomed-in views of UPS spectra of individual MoS_2_ and WS_2_ in the energy range of 17 to 14 eV (D; left dashed rectangle in Figure S16) and 5 to 0 eV (E; right dashed rectangle in Figure S16). (F) Proposed energy band diagram of the heterojunction.

Notably, the PL signal collected from the interfacial region (point 2 in Figure 5A) exhibits a pronounced and relatively broad emission peak centered at approximately 650 nm. This intermediate-energy emission is significantly shifted from the excitonic peaks of the individual monolayers. Additionally, the PL intensity map at 650 nm (Figure 5C) reveals that the enhanced PL response is highly localized at the phase boundary. We attribute the ∼1.91 eV (∼650 nm) emission predominantly to direct interfacial recombination, namely radiative recombination of electrons in the conduction band of MoS_2_ with holes in the valence band of WS_2_. This process is promoted by excitonic recombination centers induced by the strong built-in electric field at the atomically sharp interface.^8^ Consequently, the interface acts as a localized recombination site for photogenerated electron–hole pairs. Although SPVM measurements reveal long-range charge separation across the heterojunction, the PL results indicate pronounced localized recombination at the interface, suggesting that interfacial recombination constitutes an intrinsic energy-loss channel in the in-plane MoS_2_–WS_2_ heterojunction. Similar interface-related emissions have also been reported in other two-dimensional heterostructures, highlighting the generality of this phenomenon.^26^

To gain deeper insight into the driving force for charge separation in the heterojunction, UPS was employed to investigate the electronic band structures of MoS_2_ and WS_2_ (Figure S16). The work functions of MoS_2_ and WS_2_ were determined by measuring the Fermi level and the secondary electron cutoff, and are defined as the energy difference between the vacuum level (*E*_VAC_) and the Fermi level (*E*_F_). The measured work functions are 5.49 eV for MoS_2_ and 5.34 eV for WS_2_ (Figure 5D), indicating that the Fermi level of WS_2_ is approximately 0.15 eV higher than that of MoS_2_. The relative positions of the valence band maxima (VBM) of MoS_2_ and WS_2_ were extracted from the UPS spectra by identifying the intersection of the tangent in the low-binding-energy region with the baseline. The VBMs of MoS_2_ and WS_2_ are located 1.59 and 1.29 eV below the Fermi level, respectively (Figure 5E), corresponding to a valence band offset of approximately 0.45 eV. The band gaps of WS_2_ and MoS_2_ were estimated from PL spectra to be 1.97 and 1.81 eV, respectively (Figure 5B). Based on the valence band offset of 0.45 eV, with the valence band of MoS_2_ lying at lower energy, the conduction band minimum (*E*_C_) of WS_2_ is inferred to be approximately 0.61 eV higher than that of MoS_2_.

The initial band structures of MoS_2_ and WS_2_ prior to contact are shown in Figure S17B. Upon formation of the heterojunction, the Fermi levels of the two materials align, resulting in a downward shift of both the conduction and valence bands of WS_2_ by approximately 0.15 eV (Figure S17C). These band offsets clearly indicate a type-II band alignment at the MoS_2_–WS_2_ interface. Under illumination, this band alignment drives photogenerated electrons toward the conduction band of MoS_2_ and photogenerated holes toward the valence band of WS_2_, leading to efficient charge separation. The experimentally observed charge transfer direction is fully consistent with the predictions based on the band diagram, while the interface simultaneously serves as a localized recombination center where electrons and holes recombine (Figure 5F).

To compare the charge separation capabilities of in-plane and vertical MoS_2_–WS_2_ heterojunctions, we synthesized vertical heterojunctions with MoS_2_ stacked on WS_2_. The formation of the vertical heterojunction was confirmed by Raman spectroscopy and Raman mapping (Figures S18 and S19). Topography and SPVM images of the vertical heterojunction (Figure 6A,B) reveal the charge transfer consistent with that observed in the lateral heterojunction, with electrons preferentially migrating toward MoS_2_ and holes toward WS_2_. Statistical analysis shows that the SPV value for the WS_2_ and MoS_2_ regions is 11 mV and −18 mV, respectively (Figure 6C). Compared with the in-plane heterojunction, the vertical heterojunction exhibits an overall weaker charge separation capacity. This difference primarily arises from fundamentally different charge separation pathways associated with the two geometries. In the vertical heterojunction, photogenerated carriers are transferred predominantly via interlayer tunneling across the van der Waals gap. By contrast, the lateral heterojunction provides a continuous in-plane lattice, allowing carriers to separate and migrate laterally within the same atomic plane, which can reduce cross-plane transport barriers and facilitate carrier separation and transport.

**Figure 6:**
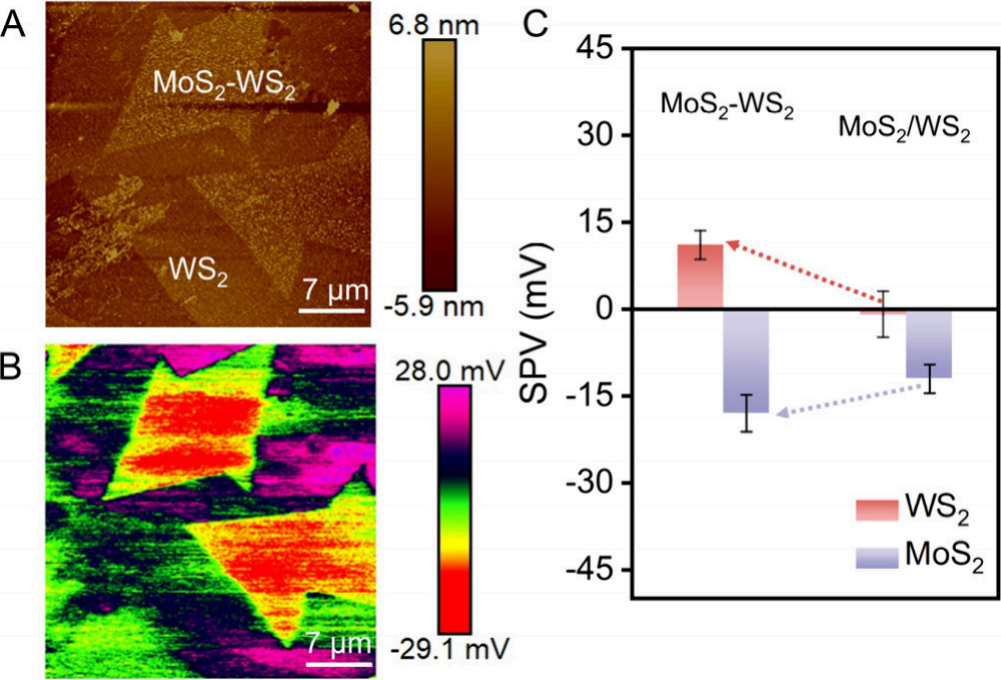
SPVM of vertical MoS_2_–WS_2_ heterojunction. (A) AFM topography image of vertical MoS_2_–WS_2_ heterojunction. (B) SPVM image of vertical MoS_2_–WS_2_ heterojunction. (C) Comparison of the mean SPV values of vertical MoS_2_–WS_2_ heterojunction to those measured in separate MoS_2_ and WS_2_ (Figures S12–S14). The error bars are the standard deviations of SPV values.

In summary, we combined photo-SECM, SPVM, and PL spectroscopy to perform in situ visualization of charge separation and recombination dynamics in monolayer MoS_2_–WS_2_ in-plane heterojunctions. The results reveal directional carrier separation, with electrons accumulating in MoS_2_ and holes in WS_2_, driven by the intrinsic type-II band alignment. However, the interface acts as a localized recombination center that limits efficient carrier extraction. For interfaces with discontinuities, the charge separation efficiency is significantly reduced. Quantitative analysis of local charge-transfer kinetics uncovers strongly asymmetric interfacial kinetics across the heterojunction at the nanoscale. Compared to vertical heterojunctions, lateral heterojunctions achieve more efficient carrier separation due to the continuity of the atomic lattice, even though the contact area between the two materials is much smaller. This work provides spatially resolved, direct visualization of charge separation in 2D type-II heterojunctions and offers critical guidelines for the rational design of high-performance lateral TMD photocatalysts and optoelectronic devices.

## Supplementary Material



## Notes

Materials and methods; supplementary SECM and AFM images, including Figures S1–S19 (PDF)
