# Supplementary material for: In Situ Imaging Reveals Efficient Charge Separation in Monolayer MoS2–WS2 Type-II Heterojunctions
Source: J Am Chem Soc. 2026 Feb 20;148(8):8417–22. doi: 10.1021/jacs.5c19244 (PMC12964524; doi:10.1021/jacs.5c19244)
Supplement: Supplementary file 1 [file ja5c19244_si_001.pdf]

Supporting Information for

In Situ Imaging Reveals Efficient Charge Separation in Monolayer MoS_2–WS_2 Type-II Heterojunctions

Qing Huang,[1,2,#] Ziyuan Wang,[3,#,*] Rujia Liu,[4,#] Hanyu Yao,[1] Chenwei Ni,[1,2] Tianyu Bo,[3,5] Shu Wu,[3,5] Fusai Sun,[1,2] Fengtao Fan,[1,*] Michael V. Mirkin[3,5,*]

[1] State Key Laboratory of Catalysis, Dalian National Laboratory for Clean Energy, iChEM, Dalian Institute of Chemical Physics, Chinese Academy of Sciences, Zhongshan Road 457, Dalian 116023, China.

[2] University of Chinese Academy of Sciences, Beijing 100049, China.

[3] Department of Chemistry and Biochemistry, Queens College-CUNY, Flushing, NY 11367, USA.

[4] College of Chemistry, Beijing Normal University, Beijing 100875, China.

[5] The Graduate Center of CUNY, New York, NY 10016, USA.

[#] These authors contributed equally.

Table of contents

Materials and Methods

Figure S1. Schematic of the synthesis process for in-plane MoS_2-WS_2 heterojunction.

Figure S2. Optical microscopic and SEM image of in-plane MoS_2-WS_2 heterojunction.

Figure S3. AFM images of in-plane MoS_2-WS_2 heterojunction.

Figure S4. Raman characterization of the in-plane MoS_2-WS_2 heterojunction.

Figure S5. Raman intensity mapping of the in-plane MoS_2-WS_2 heterojunction.

Figure S6. Atomic resolution Z-contrast STEM image showing in-plane interface in MoS_2-WS_2 heterostructure.

Figure S7. Chopped light-current transients recorded over the individual MoS_2 surface.

Figure S8. Chopped light-current transients recorded over the individual WS_2 surface.

Figure S9. Surface potential image of in-plane MoS_2-WS_2 heterojunction.

Figure S10. SPV distribution profile along the white dashed line in the in-plane MoS_2–WS_2 heterojunction.

Figure S11. SPVM images of different in-plane MoS_2–WS_2 heterojunctions.

Figure S12. SPVM images of MoS_2.

Figure S13. SPVM images of WS_2.

Figure S14. Chopped light–transient surface potential image of individual MoS_2 and WS_2.

Figure S15. PL intensity mapping of the in-plane MoS_2-WS_2 heterojunction.

Figure S16. UPS spectra of individual MoS_2 and WS_2.

Figure S17. Proposed energy band diagram of the in-plane MoS_2−WS_2 heterojunction.

Figure S18. Raman characterization of the vertical MoS_2-WS_2 heterojunction.

Figure S19. Raman intensity mapping of the vertical MoS_2-WS_2 heterojunction.

Materials and Methods

Synthesis of in-plane MoS_2-WS_2 heterojunction, MoS_2 and WS_2

MoS_2, WS_2, and in-plane MoS_2-WS_2 heterojunction were synthesized using the chemical vapor deposition (CVD) method.[1] The suspension of W and Te was prepared and spin-coated onto a SiO_2/Si substrate, where Te played a role in assisting the melting of metallic W. Sulfur powder, MoO_3, and the substrate containing W and Te were placed in a tube furnace (Anhui BEQ，BTF-1200C-Ⅱ-SL-3ZL-400C), with the temperature of the sulfur powder maintained at approximately 200°C and the temperature of MoO_3 and the SiO_2/Si substrate at around 700°C. During the reaction, argon was used for purging at a flow rate of 100 sccm. The reaction lasted for 20 minutes, with a heating rate of 10°C/min. Using this method, in-plane MoS_2-WS_2 heterojunctions were obtained. Under the same conditions, removing MoO_3 from the tube furnace resulted in the synthesis of WS_2. For MoS_2 synthesis, a clean substrate was placed downstream in the tube furnace, and the substrate temperature was controlled at approximately 850°C.[2-3]

Synthesis of vertical MoS_2–WS_2 heterojunction and sample transfer

After separately synthesizing MoS_2 and WS_2, a vertical MoS_2–WS_2 heterojunction was fabricated by transferring MoS_2 onto WS_2 using a poly(methyl methacrylate) (PMMA)-assisted transfer process to bring the two layers into intimate contact followed by PMMA removal. The same PMMA transfer procedure was used for transferring MoS_2, WS_2, and both in-plane and vertical MoS_2–WS_2 heterojunction samples. Briefly, PMMA was spin-coated onto the SiO_2/Si substrate, and the substrate was then immersed in 2 M NaOH solution to release the PMMA-supported film. The PMMA/sample film was transferred onto a new substrate, after which PMMA was removed by sequential rinsing with acetone and isopropanol, yielding the transferred sample.

Morphological characterization

The morphologies of the samples were examined by optical microscope, scanning electron microscopy (SEM, Quanta 200-FEG) and Atomic Force Microscope (AFM). High-resolution scanning transmission electron microscopy (STEM) measurements were performed using a JEM-300F microscope.

Raman characterization

Raman spectroscopy was performed under 532 nm laser excitation at room temperature with a power of 1 mW. The laser spot size was approximately 1 μm^2, and the integration time was 5 seconds. Raman mapping was carried out at room temperature using a 532 nm excitation laser. The laser power was set to 1 mW. The laser spot size was approximately 1 μm^2, and the mapping was performed with a step size of 0.5 μm.

PL characterization

PL spectra and the corresponding mapping are performed under 532 nm laser excitation with a power of 1 mW at room temperature. The spot size of the laser is about 1 μm^2. The step size for PL map is about 0.5μm.

KPFM and SPVM measurements

The surface potential (contact potential difference, CPD) of the sample was measured using Kelvin probe force microscopy (KPFM) in amplitude modulation (AM)-KPFM mode. Due to its nanometer-scale spatial resolution and millivolt-level sensitivity, KPFM enables direct imaging of surface potential under ambient conditions. To improve the signal-to-noise ratio, the lift height of the probe was set to 20 nm. The surface photovoltage microscopy (SPVM) system was built upon the KPFM setup, with the addition of a xenon lamp and a monochromator to generate

monochromatic light with tunable wavelengths. The surface photovoltage (SPV) is defined as the difference in CPD before and after illumination, i.e., SPV = $CPD_{light} - CPD_{dark}$. MoS_2, WS_2, and the lateral MoS_2–WS_2 heterojunction were excited using a 450 nm laser with an incident power of approximately 14.9 mW. Detailed information on the principles and applications of SPVM can be found in previous publications.[4-5]

In monolayer TMDs, the out-of-plane depletion region is negligible, so the measured SPV mainly originates from photoinduced quasi-Fermi-level shifts rather than electrostatic band bending. The SPV magnitude can be estimated as

$$\Delta E_F \approx kT\ln\left(1+\frac{\Delta n}{n_0}\right),$$

where Δn and n_0 are the photoexcited and equilibrium carrier densities, respectively. For typical monolayer MoS_2 and WS_2 under our illumination conditions, we estimate $\Delta n/n_0 \approx 10^{-3}$ to 10^{-2}, yielding $\Delta E_F \approx$ 5 meV. If both electron and hole quasi-Fermi levels shift by this amount, the total splitting is ~10 meV, corresponding to tens of millivolts SPV. The enhanced SPV in the heterojunction compared with the individual monolayers results from the type-II band alignment, which facilitates electron transfer to MoS_2 and hole transfer to WS_2, thereby enlarging the quasi-Fermi-level splitting, which is in agreement with our experimental values (–35 mV for MoS_2 and +20 mV for WS_2).

Photo-SECM measurements

Scanning Electrochemical Microscopy (SECM) measurements were carried out using a custom-built instrument described previously,[6] operating in a two-electrode configuration. An Ag/AgCl electrode functioned as both the reference and counter electrode, while an SECM tip served as the working electrode. The electrolyte solution comprised 1 mM ferrocenemethanol (Fc) dissolved in 0.1 M phosphate buffer (pH 7). During feedback mode experiments, the tip potential (E_T) was maintained at 0.4 V versus Ag/AgCl, and no external bias was applied to the substrate. All measurements were performed at room temperature (23 ± 2 °C) within a Faraday cage to reduce ambient noise.

For photoelectrochemical studies, the SECM instrument was combined with an optical setup supplied by Newport Corporation. Illumination was provided by a 250 W HgXe lamp powered by an OPS-A500 (500 W) power supply, with the lamp housed in a model 67005 casing and light delivered through a model 77776 fiber bundle assembly. A broad-spectrum UV–vis optical fiber (model 78277, 1 mm core) guided the light to the probe. To minimize infrared-induced heating, an FSQ-KG3 glass filter was incorporated into the optical path. A lens system focused the light onto the back side of the SECM tip, with the glass sheath of the nanoelectrode channeling illumination toward the substrate surface. Throughout all experiments, the lamp power was kept at 220 W. Light intensity at the substrate was monitored and calibrated using a PM100D power meter (Thorlabs) equipped with an S130VC silicon photodiode sensor.

UPS characterization

Ultraviolet photoelectron spectroscopy (UPS) measurements were performed using a He I (21.22 eV) light source. The energy step size was set to 0.020 eV, and a total of 1251 energy steps were collected during the measurement. These settings allowed for detailed characterization of the sample's valence band structure and electronic properties.

COMSOL simulation

Finite-element simulations of the SECM responses were performed using a commercial software package COMSOL Multiphysics 5.4 with a 3D model. The mass transport processes in the SECM experiments were described by the Nernst-Planck equation:

$$J_i =- D_i \nabla c_i \tag{S1}$$

where J_i is the total flux, D_i, and c_i are the diffusion coefficient and concentration of species i, respectively. Electron transfer (ET) at the tip was described by the Butler–Volmer expressions:

$$J_R = k^0 c_O e^{-\alpha f(E-E^{0'})} - k^0 c_R e^{(1-\alpha)f(E-E^{0'})} \tag{S2}$$

$$J_O =- k^0 c_O e^{-\alpha f\left(E-E^{0'}\right)} + k^0 c_R e^{(1-\alpha)f(E-E^{0'})} \tag{S3}$$

where k^0 is the standard rate constant and $E^{0'} = 0.2$ V is formal potential for the ET process. $f =$ zF/RT, and α is the transfer coefficient. (Here $O \equiv Fc^+$, $R \equiv Fc$.)

Substrate treatment and light/dark definition. In the simulations the substrate was treated as a metallic conductor so that heterogeneous surface reactions could be imposed on selected geometric regions. This metallic model was not intended to reproduce semiconductor photophysics; rather, it was used to (i) extract interfacial kinetic parameters from experimental approach curves and (ii) validate that the same parameters reproduce spatial imaging features.

Under illumination, the substrate reactions were prescribed as simple first-order surface processes:

On the MoS_2 side (inner triangular region):

$$O \overset{k_{s1}}{\longrightarrow} R \tag{S4}$$

On the WS_2 side (outer triangular ring):

$$R \overset{k_{s2}}{\longrightarrow} O \tag{S5}$$

Where k_{s1} and k_{s2} are the substrate kinetic constants to be determined. The dark condition was implemented by removing surface reactions on the metallic substrate.

The resulting tip current was obtained by integrating the flux at the tip surface:

$$i = \int F J_{\mathrm{R}} \mathrm{d}S \tag{S6}$$

where F is the Faraday constant, and S is the electrode surface.

Geometry, parameters, and boundaries.

The tip was modeled as a disk with conductive radius a = 70 nm and glass sheath ratio RG = 2.5 (ratio of glass radius to conductive radius). Diffusion coefficients were D_{Fc}= 7.9 × 10^{-6} cm^2 s^{-1} and D_{Fc^+} = 4.5 × 10^{-6} cm^2 s^{-1}. The tip potential was E_T= 0.4 V with $E°'_{Fc}$ = 0.2 V. The far-field boundary was fixed at c^{∞} = 1 mM for both species; non-reactive boundaries were set to zero flux. The Fc concentration map in Figure 2D corresponds to the substrate surface (z = 0 plane).

Fitting workflow and validation.

1. The experimental approach curve of MoS_2 in the dark (Figure 2A, solid line) was first fitted with the simulation (red symbols) to determine the geometric parameters $a = 70$ nm and $RG = 2.5$.

2. On this basis, the approach curves of MoS_2 and WS_2 under illumination (Figures 2B–C, solid lines; simulations shown as red symbols) were fitted, yielding $k_{s1} = 0.6$ cm s^{-1} (Fc^+ reduction on MoS_2) and $k_{s2} = 0.008$ cm s^{-1} (Fc oxidation on WS_2). The two values differ by nearly two orders of magnitude, consistent with directional charge separation in the lateral heterojunction.

3. Using the same set of k_{s1}/k_{s2}, forward simulations were performed to obtain the Fc concentration distribution at the substrate surface (Figure 2D, showing depletion/accumulation near the reactive regions) and the lateral tip scan along the dashed arrow in Figure 2D (Figure 2E). The simulated spatial features agree well with the experimental photo-SECM images (Figures 1B–C).

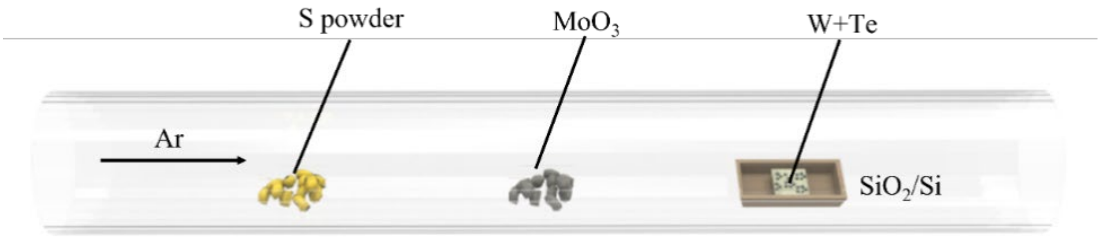

Figure S1. Schematic of the synthesis process for in-plane MoS_2-WS_2 heterojunction.

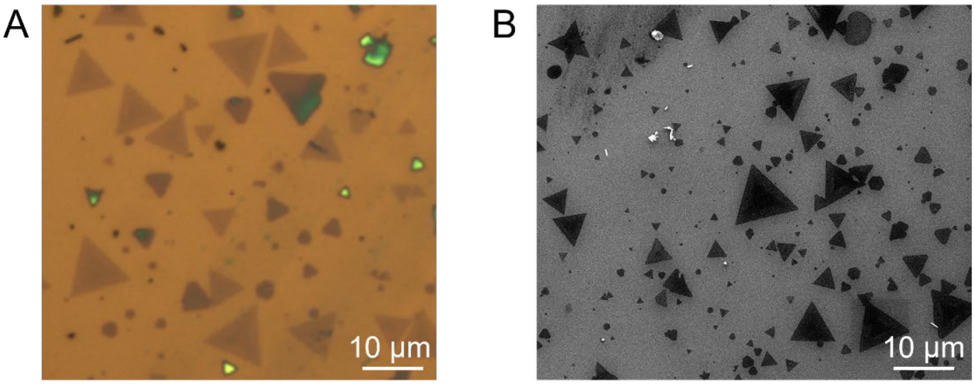

Figure S2. (A) Optical microscopic image and (B) scanning electron microscopy (SEM) image of in-plane MoS_2-WS_2 heterojunctions. The inner darker region represents MoS_2, while the outer lighter region corresponds to WS_2.

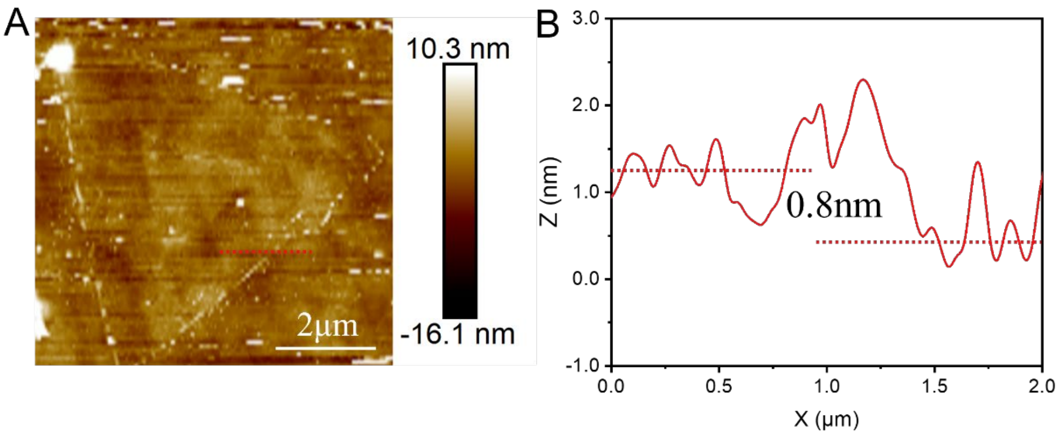

Figure S3. (A) AFM topography image of in-plane MoS_2-WS_2 heterojunction (same heterojunction as in Figure 4A). (B) Height profile along the red line in (A), showing the height of about 0.8 nm.

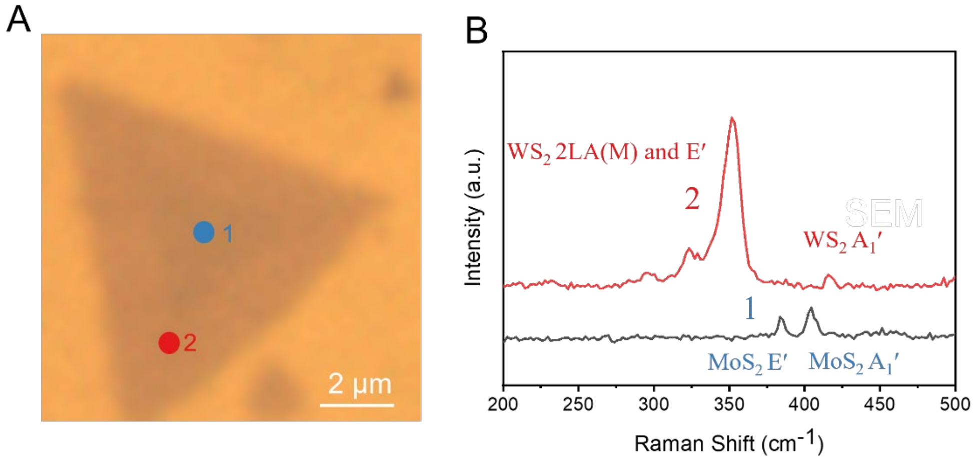

Figure S4. (A) Optical microscopic image of the in-plane MoS_2-WS_2 heterojunction used for Raman characterization. (B) Raman spectra taken from the two points marked in (A).

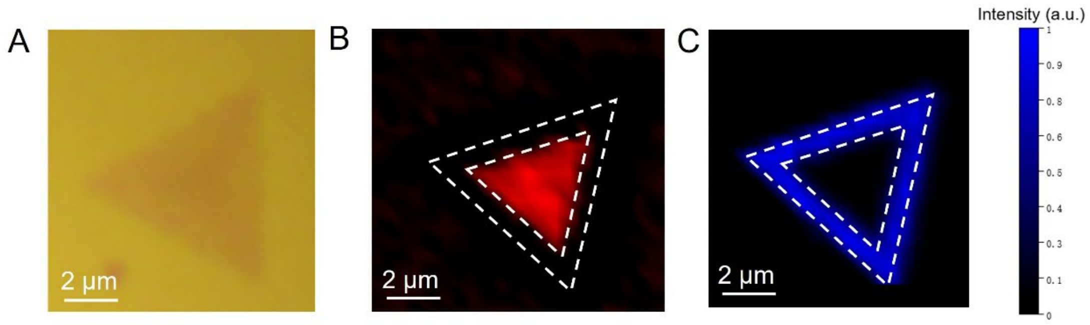

Figure S5. (A) Optical microscopic image of the in-plane MoS_2-WS_2 heterojunction used for Raman intensity mapping. (B) Raman intensity mapping at 381 cm^{-1} and (C) at 351 cm^{-1}, confirming the lateral spatial distribution, where MoS_2 occupies the inner domain and WS_2 forms the outer domain.

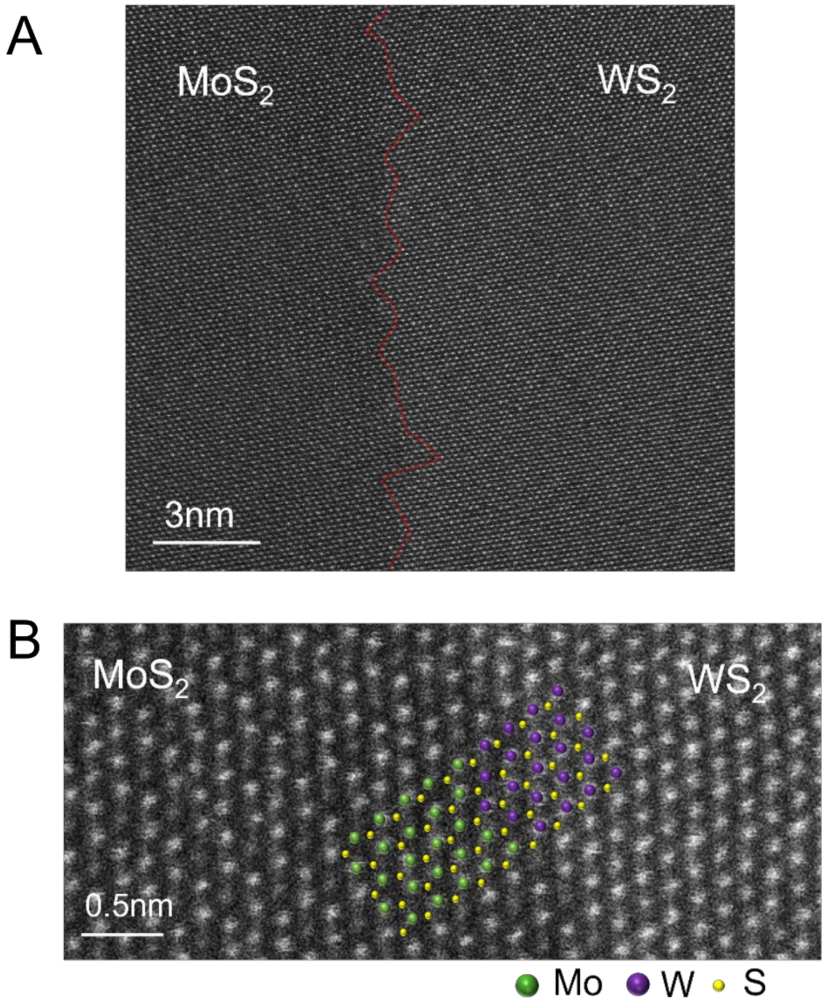

Figure S6. Atomic-resolution Z-contrast STEM images showing the in-plane interface of the MoS_2–WS_2 heterostructure. The atomically sharp interface along the zigzag direction is indicated by the red dashed line in panel A. In panel B, selected atoms in specific regions are labeled for clarity.

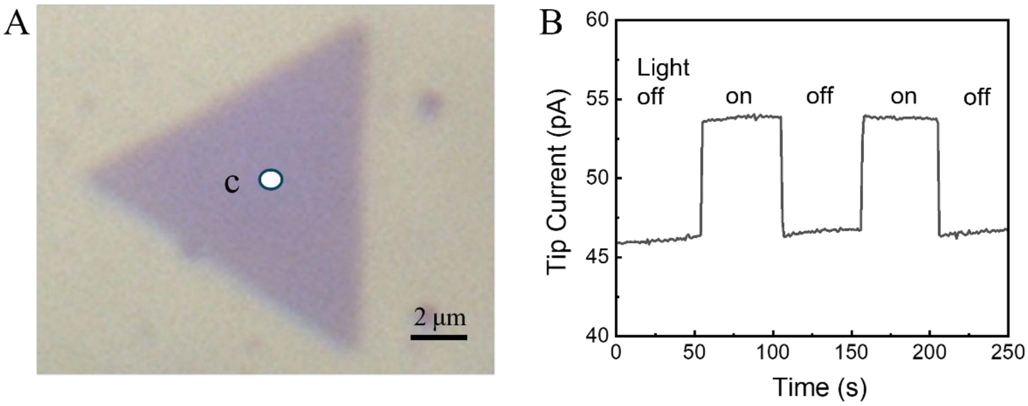

Figure S7. (A) Optical microscopic image of a MoS_2 triangle and (B) chopped light-current transients recorded over point **c** on the MoS_2 surface.

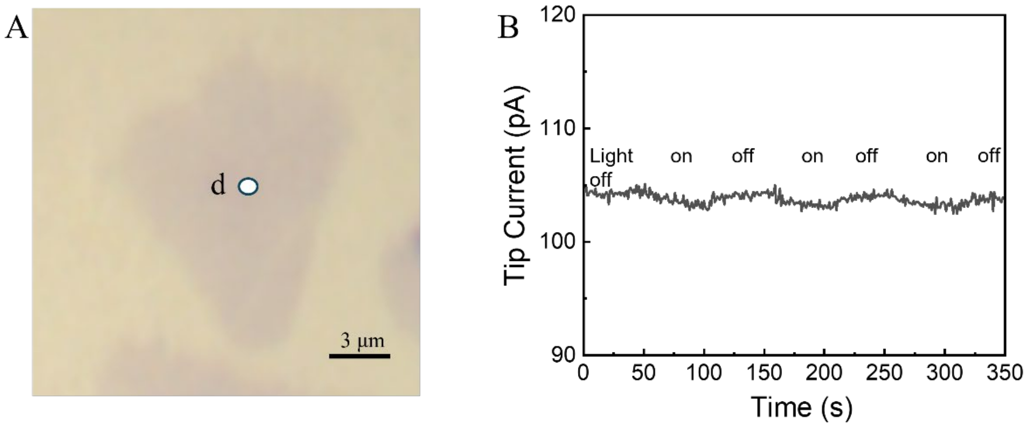

Figure S8. (A) Optical micrograph of a WS_2 triangle and (B) chopped light-current transients recorded over point **d** on the WS_2 surface.

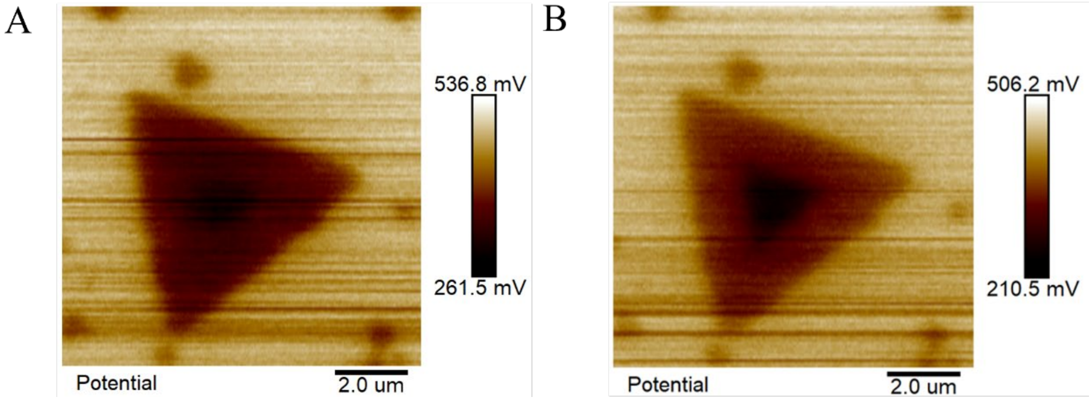

Figure S9. Contact potential difference (CPD) image in the dark (A) and under 450 nm light illumination (B).

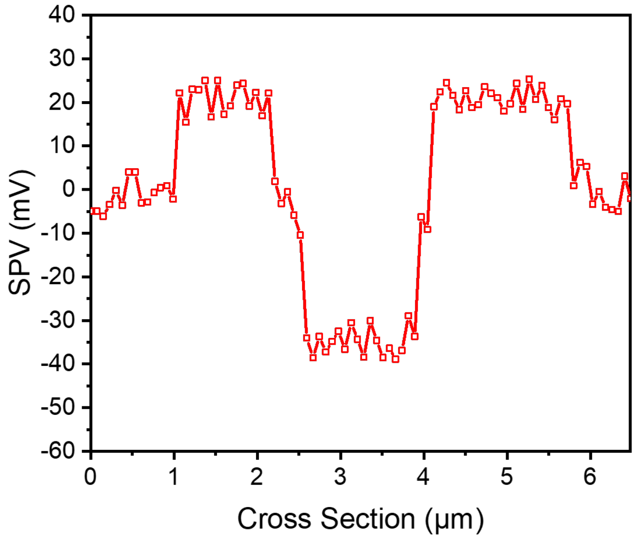

Figure S10. SPV distribution profile measured in the in-plane MoS_2–WS_2 heterojunction along the white dashed line in Figure 4B.

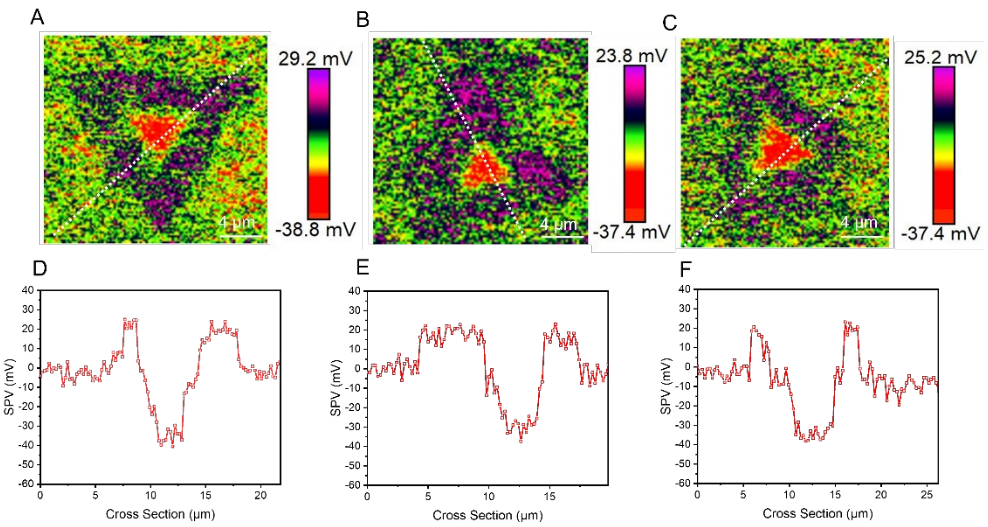

Figure S11. (A-C) SPVM images of different MoS_2–WS_2 in-plane heterojunctions. (D–F) SPV distribution profiles measured along the white dashed lines in panels A–C, respectively.

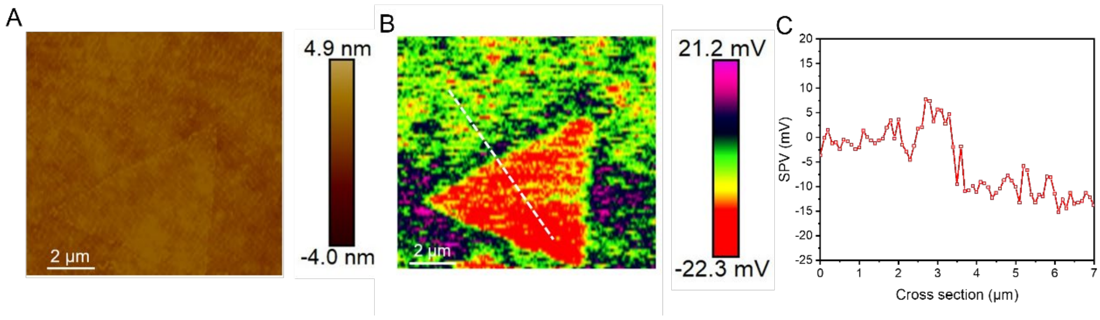

Figure S12. SPVM of MoS_2. (A) AFM topography image of MoS_2. (B) SPVM image of MoS_2. (C) The specific SPV values measured along the white dashed line indicated in (B), giving approximately an SPV value of −12 mV for the individual MoS_2 sample.

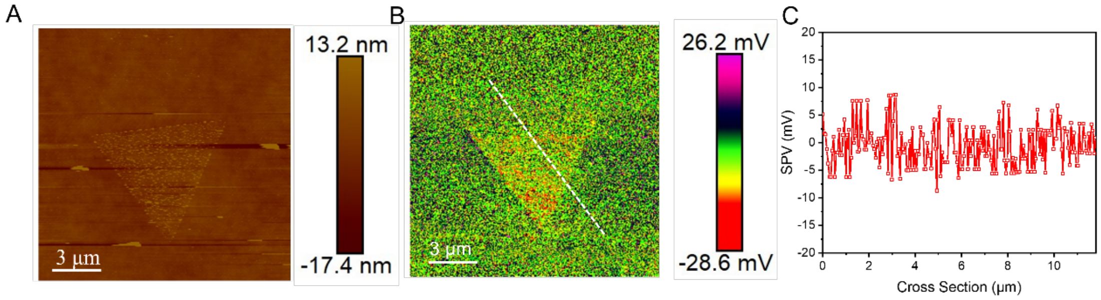

Figure S13. SPVM of WS_2. (A) AFM topography image of WS_2. (B) SPVM image of WS_2. (C) The specific SPV values measured along the white dashed line indicated in (B), giving an SPV value of about −1 mV for the individual WS_2 sample.

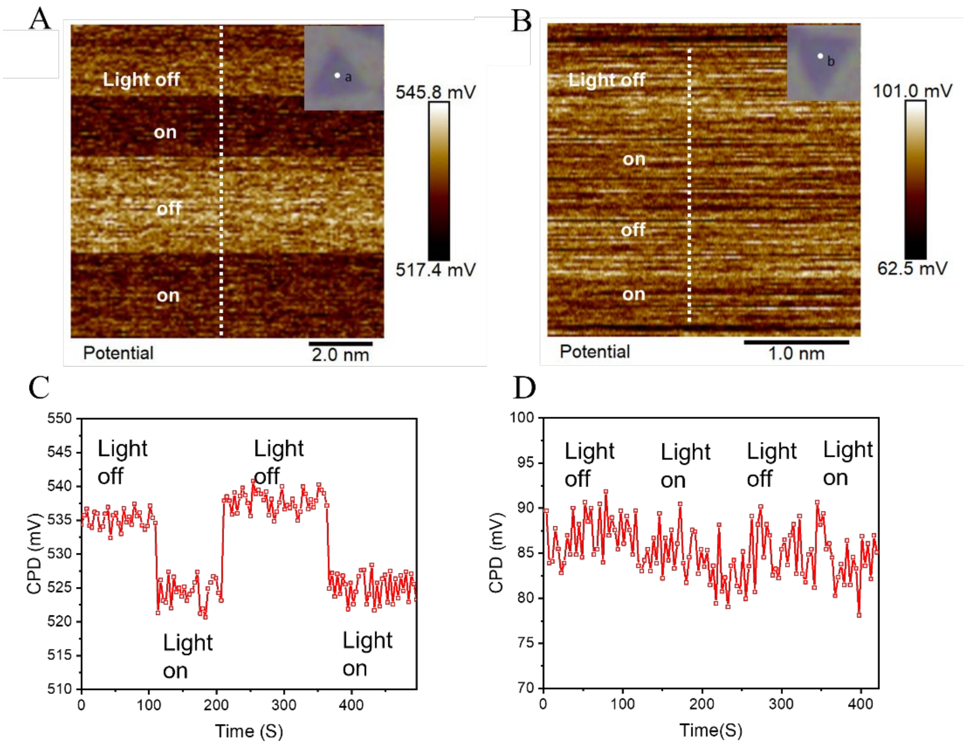

Figure S14. (A,B) CPD images recorded at point **a** on the MoS_2 triangle marked in the inset (A) and point **b** on the WS_2 surface marked in the inset (B) while intermittently turning the light on and off. (C) and (D) CPD values extracted along the white dashed line in (A) and (B), respectively.

We performed alternating light on/off measurements of the contact potential difference (CPD) at individual points on the MoS_2 and WS_2 samples, recording the CPD values under illumination and in the dark, respectively. The surface photovoltage (SPV) was obtained by subtracting the CPD in the dark from that under illumination, and the measured SPV values are consistent with the results shown in Figures S12 and S13.

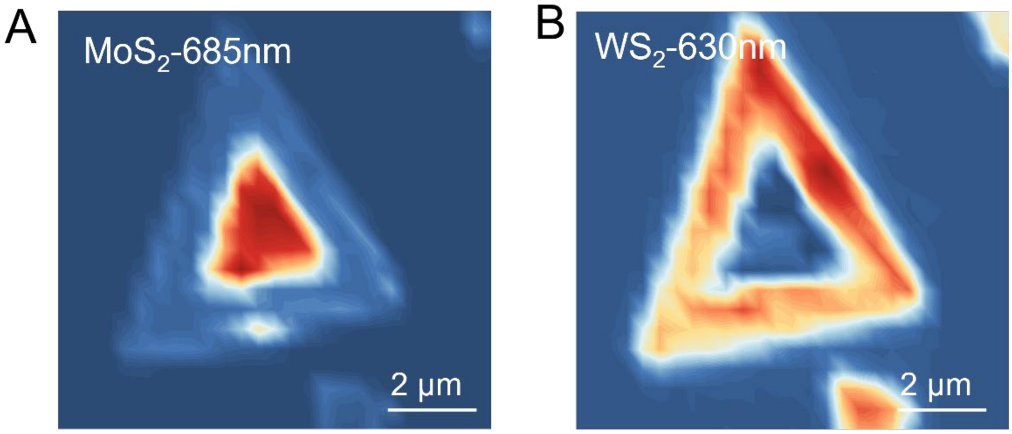

Figure S15. PL intensity mapping of the MoS_2-WS_2 lateral heterojunction recorded at detection wavelengths of 685 nm (A) and 630 nm (B), corresponding to the A-exciton emissions of MoS_2 and WS_2, respectively.

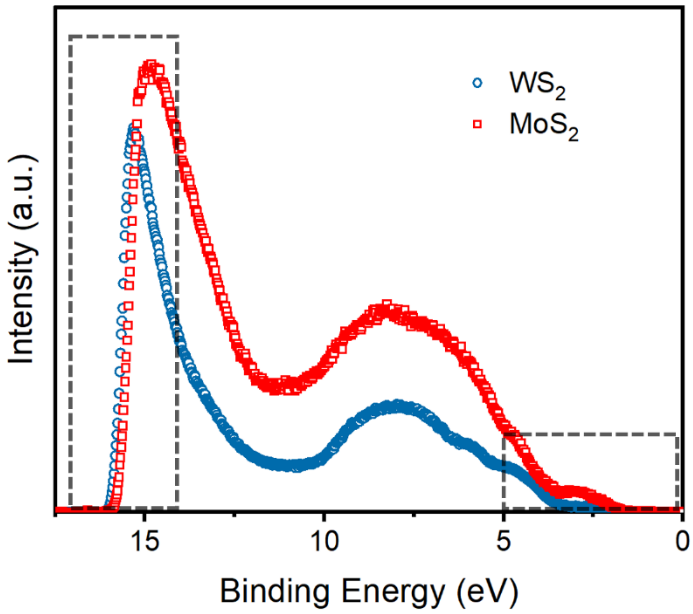

Figure S16. UPS spectra of separate MoS_2 and WS_2 samples. A magnified view of the of portions of these spectra inside the left and right dashed rectangular regions are shown in Figures 5D and 5E, respectively.

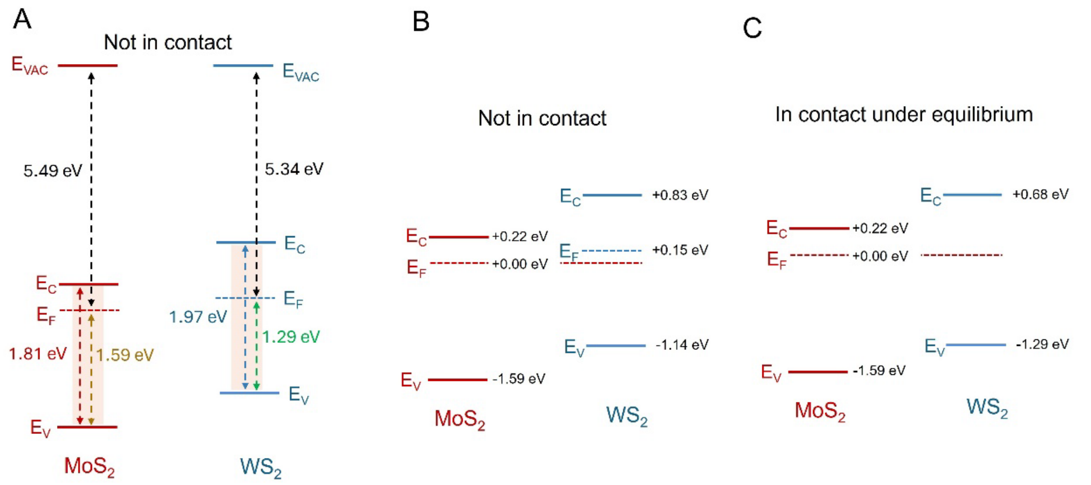

Figure S17. Proposed energy band diagrams of the MoS_2−WS_2 heterojunction. (A) The initial band structures of MoS_2 and WS_2 (not in contact) derived from UPS and PL data. (B) Same as in (A), but with the Fermi level of MoS_2 set as the zero reference. (C) Band structures of MoS_2 and WS_2 in contact under equilibrium conditions.

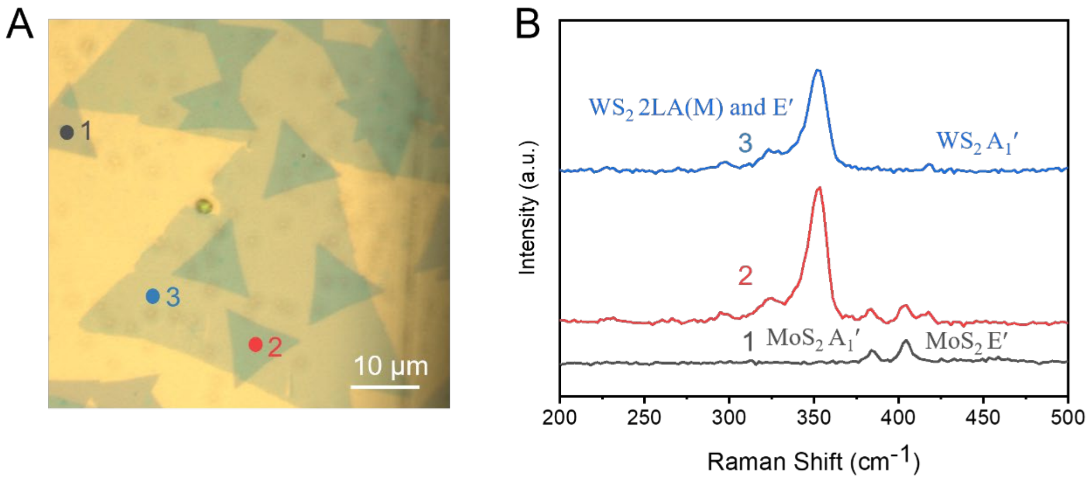

Figure S18. (A) Optical microscopic image of the vertical MoS_2-WS_2 heterojunction used for Raman characterization. (B) Raman spectra taken from the three points marked in (A).

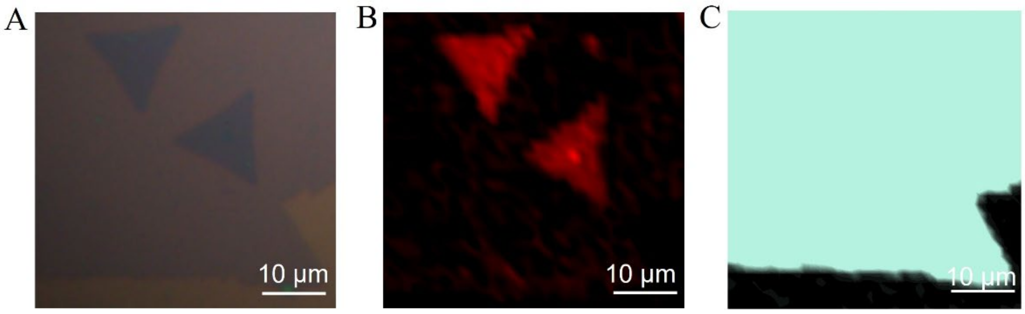

Figure S19. (A) Optical microscopic image of the vertical MoS_2-WS_2 heterojunction used for Raman intensity mapping. (B) Raman intensity mapping at 381 cm^{-1} and (C) at 351 cm^{-1}, showing the small triangular region at the top corresponds to MoS_2, while the large area underneath corresponds to WS_2.

1. Gong, Y.; Lin, J.; Wang, X.; Shi, G.; Lei, S.; Lin, Z.; Zou, X.; Ye, G.; Vajtai, R.; Yakobson, B. I.; Terrones, H.; Terrones, M.; Tay, Beng K.; Lou, J.; Pantelides, S. T.; Liu, Z.; Zhou, W.; Ajayan, P. M., Vertical and in-plane heterostructures from WS_2/MoS_2 monolayers. *Nat. Mater.* **2014,** *13* , 1135-1142.
2. Najmaei, S.; Liu, Z.; Zhou, W.; Zou, X.; Shi, G.; Lei, S.; Yakobson, B.; Idrobo, J.; Ajayan, P.; Lou, J., Vapour phase growth and grain boundary structure of molybdenum disulphide atomic layers. *Nat. Mater.* **2013,** *12*, 754-759.
3. van der Zande, A.; Huang, P.; Chenet, D.; Berkelbach, T.; You, Y.; Lee, G.; Heinz, T.; Reichman, D.; Muller, D.; Hone, J.; Grains and grain boundaries in highly crystalline monolayer molybdenum disulphide. *Nat. Mater.* **2013,** *12*, 554-561.
4. Chen, R.; Fan, F.; Dittrich, T.; Li, C., Imaging photogenerated charge carriers on surfaces and interfaces of photocatalysts with surface photovoltage microscopy. *Chem. Soc. Rev.* **2018,** *47*, 8238-8262.
5. Chen, R.; Fan, F.; Li, C., Unraveling Charge-Separation Mechanisms in Photocatalyst Particles by Spatially Resolved Surface Photovoltage Techniques. *Angew. Chem. Int. Ed.* **2022,** *61*, e202117567.
6. Wang, Z.; Huang, Q.; Ni, C.; Bo, T.; Fan, F.; Mirkin, M., Photoelectrochemical Imaging of Charge Separation between MoS_2 Triangles and Insulating SiO_2 Support. *J. Am. Chem. Soc.* **2025**, 147, 14924-14929.

MoS2-WS2-3D-A

Contents

1. Global Definitions 28

1.1. Parameters 28

2. Component 1 29

2.1. Definitions 29

2.2. Geometry 1 29

2.3. Transport of Diluted Species 30

2.4. Mesh 1 31

3. Study 1 32

3.1. Parametric Sweep 32

3.2. Stationary 32

4. Results 33

4.1. Data Sets 33

4.2. Derived Values 34

4.3. Plot Groups 35

1 Global Definitions

Date	Oct 30, 2024 3:02:13 PM

GLOBAL SETTINGS

Name	MoS2-WS2-3D-A.mph
Path	D:\simulation-other\ziyuan\ZIYUAN\MoS2-WS2-3D-A.mph
Version	COMSOL Multiphysics 5.6 (Build: 280)

USED PRODUCTS

COMSOL Multiphysics
CAD Import Module
Chemical Reaction Engineering Module

COMPUTER INFORMATION

CPU	Intel64 Family 6 Model 170 Stepping 4, 22 cores
Operating system	Windows 10

1.1 PARAMETERS

PARAMETERS 1

Name	Expression	Value	Description
at	70[nm]	7E−8 m	tip radius
as	30[µm]	3E−5 m	substrate radius
RG	2.5	2.5	RG
d	600[nm]	6E−7 m	distance
DO	4.5e-10[m*m/s]	4.5E−10 m²/s	diffusion coefficient
DR	7.9e-10[m*m/s]	7.9E−10 m²/s	diffusion coefficient
kt	6.8[cm/s]	0.068 m/s	tip kinetics
ks1	0.5[cm/s]	0.005 m/s	sub kinetics(MoS2)
cbO	0[mM]	0 mol/m³	bulk concentration
cbR	1[mM]	1 mol/m³	bulk concentration
F	96485[C/mol]	96485 C/mol	
E0	0.2[V]	0.2 V	standard potential
ET	0.4[V]	0.4 V	tip potential
ks2	0.008[cm/s]	8E−5 m/s	sub kinetics(WS2)

2 Component 1

SETTINGS

Description	Value
Avoid inverted elements by curving interior domain elements	Off

2.1 DEFINITIONS

2.1.1 Coordinate Systems

Boundary System 1

Coordinate system type	Boundary system
Tag	sys1

COORDINATE NAMES

First	Second	Third
t1	t2	n

2.2 GEOMETRY 1

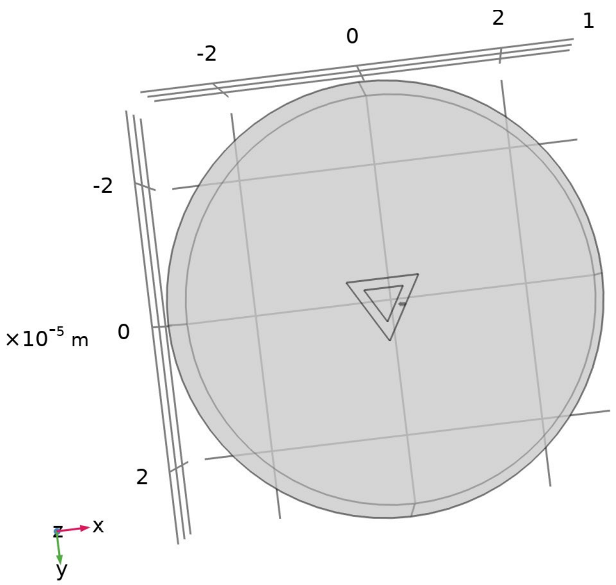

Geometry 1

UNITS

Length unit	m
Angular unit	deg

2.3 TRANSPORT OF DILUTED SPECIES

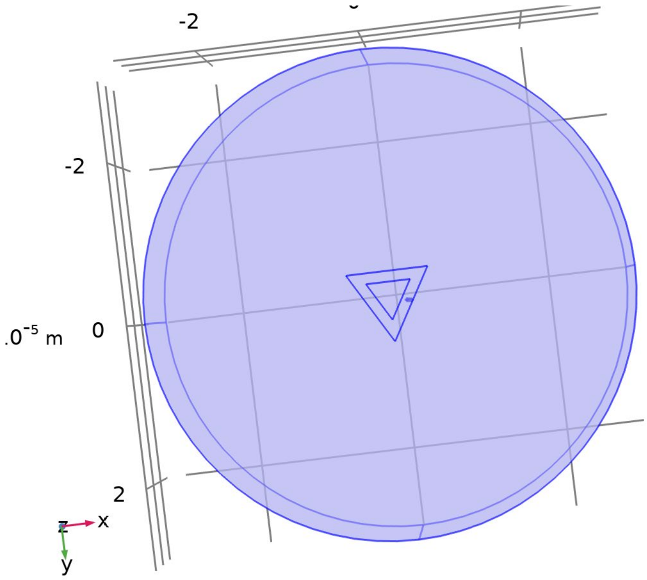

Transport of Diluted Species

EQUATIONS

$$\nabla \cdot \mathbf{J}_i = R_i$$
$$\mathbf{J}_i = -D_i \nabla c_i$$

FEATURES

**Name**	**Level**
Transport Properties 1	Domain
No Flux 1	Boundary
Initial Values 1	Domain
Concentration 1	Boundary
Flux 1	Boundary
Flux 2	Boundary
Flux 3	Boundary

2.4 MESH 1

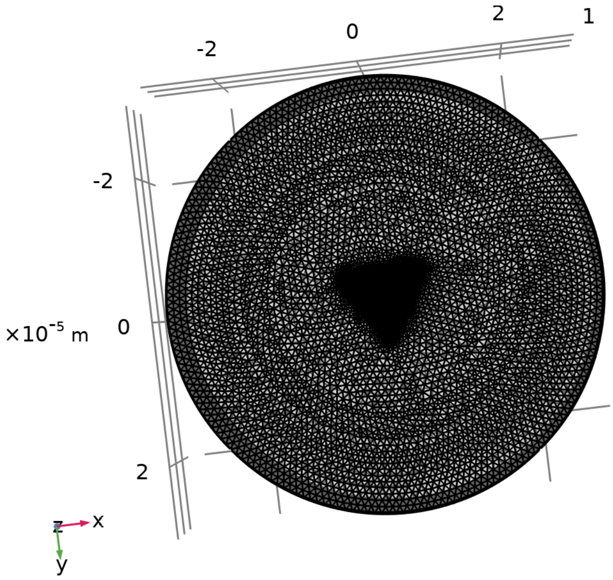

Mesh 1

3 Study 1

COMPUTATION INFORMATION

Computation time	30 s

3.1 PARAMETRIC SWEEP

Parameter name	Parameter value list	Parameter unit
d	range(2,-0.07894736842105263,0.5) range(0.5,-0.025789473684210525,0.01)	um

STUDY SETTINGS

Description	Value
Sweep type	All combinations
Parameter name	d
Unit	um

PARAMETERS

Parameter name	Parameter value list	Parameter unit
d (distance)	range(2,-0.07894736842105263,0.5) range(0.5,-0.025789473684210525,0.01)	um

3.2 STATIONARY

STUDY SETTINGS

Description	Value
Include geometric nonlinearity	Off

PHYSICS AND VARIABLES SELECTION

Physics interface	Discretization
Transport of Diluted Species (tds)	physics

MESH SELECTION

Geometry	Mesh
Geometry 1 (geom1)	mesh1

4 Results

4.1 DATA SETS

4.1.1 Study 1/Solution 1

SOLUTION

Description	Value
Solution	Solution 1
Component	Component 1 (comp1)

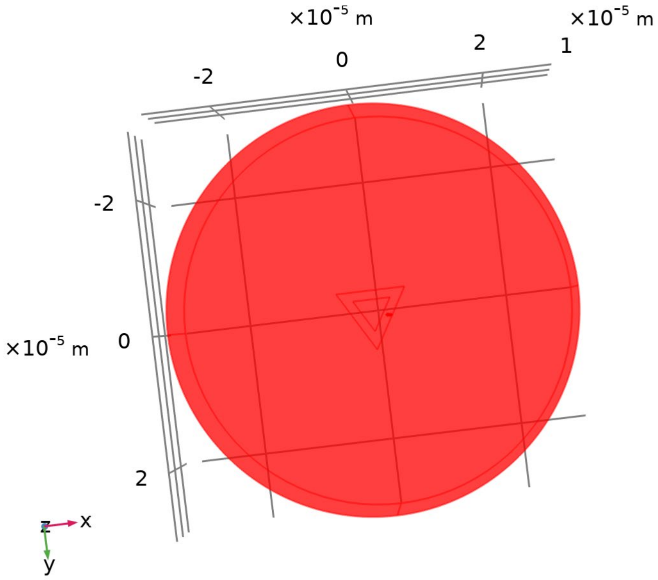

Dataset: Study 1/Solution 1

4.1.2 Study 1/Parametric Solutions 1

SOLUTION

Description	Value
Solution	Parametric Solutions 1
Component	Component 1 (comp1)

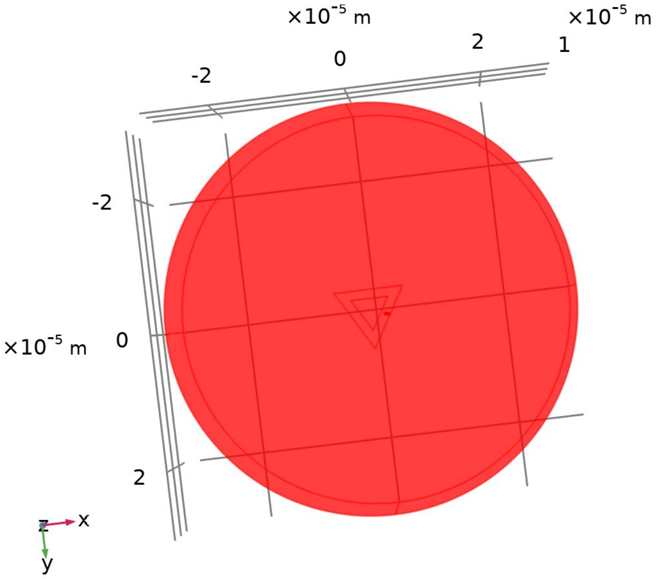

Dataset: Study 1/Parametric Solutions 1

4.2 DERIVED VALUES

4.2.1 Surface Integration 1

DATA

Description	Value
Dataset	

EXPRESSIONS

Expression	Unit	Description
tds.ntflux_cR*F	A	

INTEGRATION SETTINGS

Description	Value
Integration order	4

4.3 PLOT GROUPS

4.3.1 2D Plot Group 5

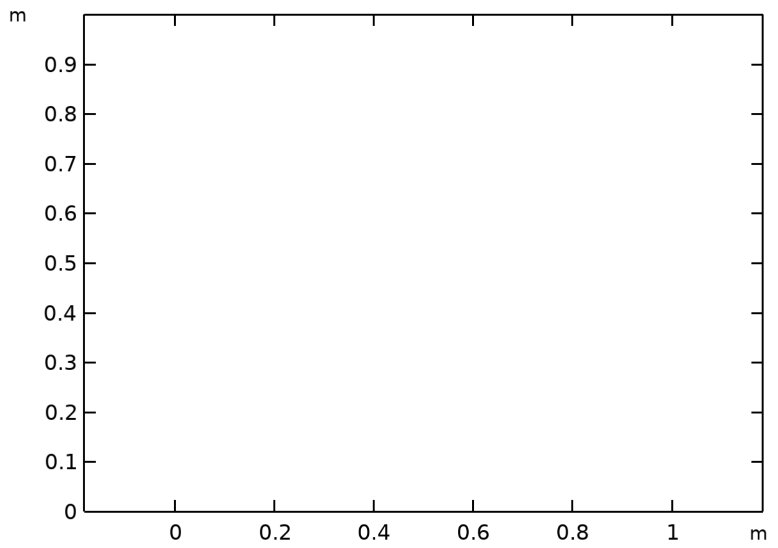

4.3.2 Concentration, O, Streamline (tds)

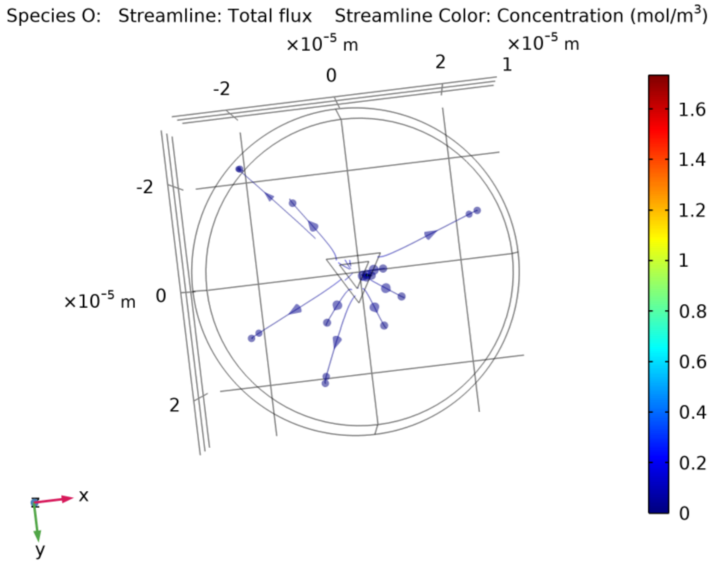

Species O: Streamline: Total flux Streamline Color: Concentration (mol/m^3)

4.3.3 Concentration, O, Surface (tds)

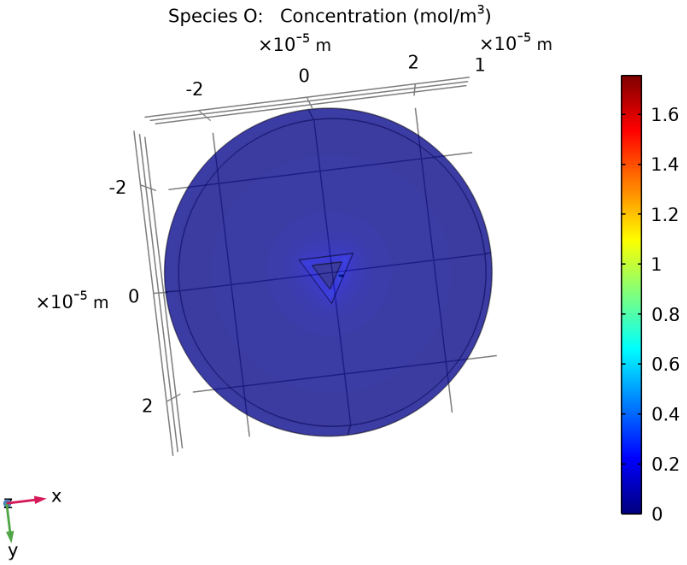

Species O: Concentration (mol/m^3)

4.3.4 Concentration, R, Streamline (tds)

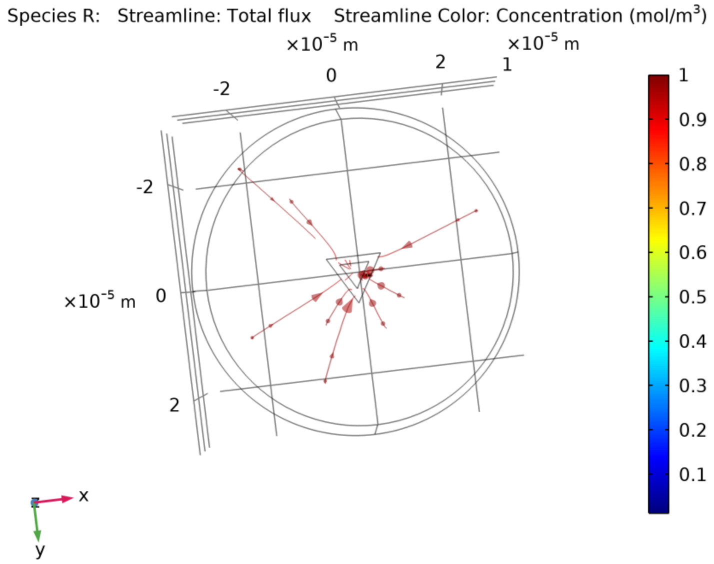

Species R: Streamline: Total flux *Streamline Color: Concentration (mol/m^3)*

4.3.5 Concentration, R, Surface (tds)

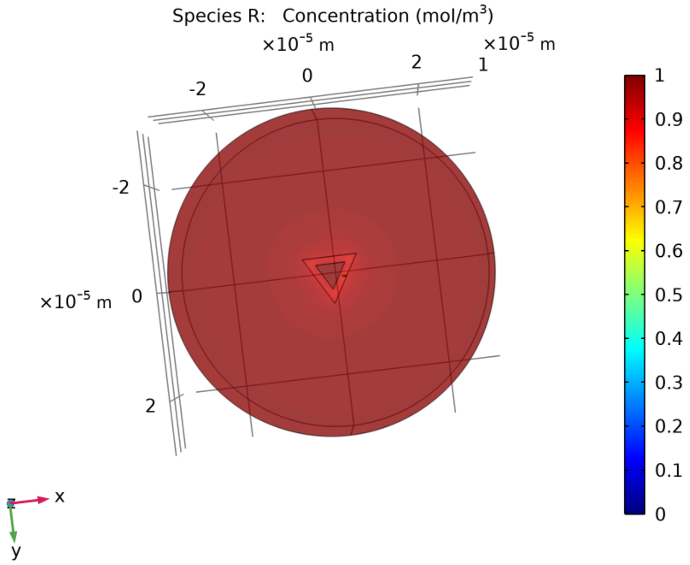

Species R: Concentration (mol/m^3)
